# Disregulations of PURPL and MiR-338-3p Could Serve As Prognosis Biomarkers for Epithelial Ovarian Cancer

**DOI:** 10.7150/jca.61327

**Published:** 2021-07-25

**Authors:** Ruitao Zhang, Tingting He, Huirong Shi, Cao Yuan, Feng Wei, Zheying Liu, Wenwen Wang

**Affiliations:** Department of Gynecology, First Affiliated Hospital, Zhengzhou University, NO.1 East Jianshe Road, Erqi District, Zhengzhou, Henan, 450052 P.R. China

**Keywords:** biomedical database, epithelial ovarian cancer, PURPL, miR-338-3p, prognosis

## Abstract

**Objective:** The present study aimed to explore the expressions of long noncoding RNA (lncRNA) p53 upregulated regulator of p53 levels (PURPL) in different ovarian tissues, and to evaluate the significance of disregulations of PURPL and microRNA-338-3p (miR-338-3p) in epithelial ovarian cancer (EOC).

**Methods:** The expressions of PURPL in ovarian cancer, the relations between PURPL and the prognosis of ovarian cancer, and the relation between PURPL and miR-338-3p were queried in multiple biomedical databases. Real-time PCR was performed to detect the expressions of PURPL in different ovarian tissues. Logistic regression analysis was used to analyze the risk factors of recurrence and death. Kaplan-Meier analysis was implemented to evaluate the relations between PURPL and miR-338-3p expressions and the survival of ovarian cancer.

**Results:** PURPL could target miR-338-3p, PURPL were upregulated in ovarian cancer tissues, upregulation of PURPL in ovarian cancer was negatively related with the recurrence free survival (RFS) and overall survival (OS), which were indicated by biomedical databases query. Our data showed upregulations of PURPL were noted in ovarian cancer tissues. Higher expressions of PURPL were associated with more advanced FIGO stage and developed lymph node metastasis in epithelial ovarian cancer. Upregulation of PURPL was related with the recurrence (P=0.002, OR=21.482, 95%CI: 3.457~94.251) and death (P=0.004, OR=35.643, 95%CI: 2.453~84.359) of ovarian cancer patient. PURPL expressions were negatively correlated to miR-338-3p expressions in different ovarian tissues (r = -0.968, P<0.0001). Poor RFS (χ^2^=19.410, P=0.0002) and OS (χ^2^=17.600, P=0.0005) were found in patients with high level PURPL and low level miR-338-3p expressions.

**Conclusions:** Upregulation of PURPL and downregulation of miR-338-3p were related with the poor RFS and OS of ovarian cancer, which indicated disregulations of PURPL and miR-338-3p could serve as prognosis biomarkers for epithelial ovarian cancer.

## Introduction

Although gene target therapy and immunity therapy bring new hope to the treatments of ovarian cancer patients, ovarian cancer still is the most lethal malignancy in female genital tract tumors 1. Even though considerable progress in molecular mechanism research of the growth and metastasis of ovarian cancer has been made, further research is still needed. Emerging reports have shown that noncoding RNAs, including long noncoding RNAs (lncRNAs) and micro noncoding RNAs (miRNAs), play important roles in the growth and metastasis of human malignant tumors 2-6. LncRNAs were reported to be important regulatory molecules that could regulate the growth and metastasis of malignant tumors via targeting and sponging downstream miRNAs 7-8.

Our previous data indicated miR-338-3p was downregulated and MACC1 was upregulated in epithelial ovarian cancer, and was related with poor prognosis of ovarian cancer 9. We tried to explore the upstream regulated lncRNAs of miR-338-3p to analyze their roles in the development and progression of ovarian cancer in present study. Biomedical database queries showed that lncRNA p53 upregulated regulator of p53 levels (PURPL) could target miR-338-3p. PURPL, also known as LINC01021, was detected to be upregulated in gastric cancer tissue, and was related to tumor size and histopathological grade, which indicated poor clinical outcomes 10. Rare reports were involved in the relations between PURPL and ovarian cancer.

In the present study, we tried to query the expression of PURPL and its relation with the prognosis of ovarian cancer in biomedical databases. Then, real time PCR was performed to confirm the expressions of PURPL in different collected ovarian tissues, and to explore the significance of PURPL and miR-338-3p in the prognosis of ovarian cancer.

## Methods

### Biomedical database query

Multiple biomedical databases, including ENCORI 11, miRcode (http://www.mircode.org/) and LncBase Predicted 12 were used to predict the relation between PURPL and miR-338-3p. Pubmed (https://pubmed.ncbi.nlm.nih.gov/), cBioPortal 13, lncRNASNP2 14, GEPIA 15 were used to query the expressions of PURPL in normal and malignant human tissues. Kaplan-Meier Plotter 16 was used to analyze the relations between PURPL and the prognosis of ovarian cancer.

### Tissue specimens

All enrolled fresh different ovarian tissue specimens of 105 patients were collected and saved in the First Affiliated Hospital of Zhengzhou University. There were 20 cases of normal ovarian tissues, 20 cases of benign ovarian epithelial tumor tissues, and 65 cases of primary lesions of newly diagnosed epithelial ovarian cancer. Complete detailed clinic parameters and detailed criteria for the inclusion and exclusion of patients enrolled were described in detail in our previous report 9. Consent from patients or family members and approval by the Ethics Committee of the First Affiliated Hospital of Zhengzhou University was received for collection and use of all tissue samples.

### Real time PCR

Total RNA isolation, cDNA reverse-synthesis and SYBR Green PCR assay procedures were described in our previous report 9. The primers used in PCR were synthesized by Shanghai Sangon Biotech Co., Ltd. The primer sequences were shown in Table 1. Each PCR experiment was performed three times independently, and the relative expression values were expressed by 2^-ΔΔCt^ method.

### Statistical Analysis

Average values were expressed as mean ± standard deviation, and GraphPad Prism 7 software package was used to analyze data. One-way ANOVA, Non-parametric test, Independent sample T test, Binary logistic regression analysis, Spearman correlation test and Kaplan-Meier analysis were performed respectively to explore the expression difference, relations with clinicopathologic characteristics, risk factors of recurrence and death analysis, expression interrelationship analysis, and survival analysis. Difference was considered significant when P value was less than 0.05.

## Results

### Biomedical database query

**Figure 1:**
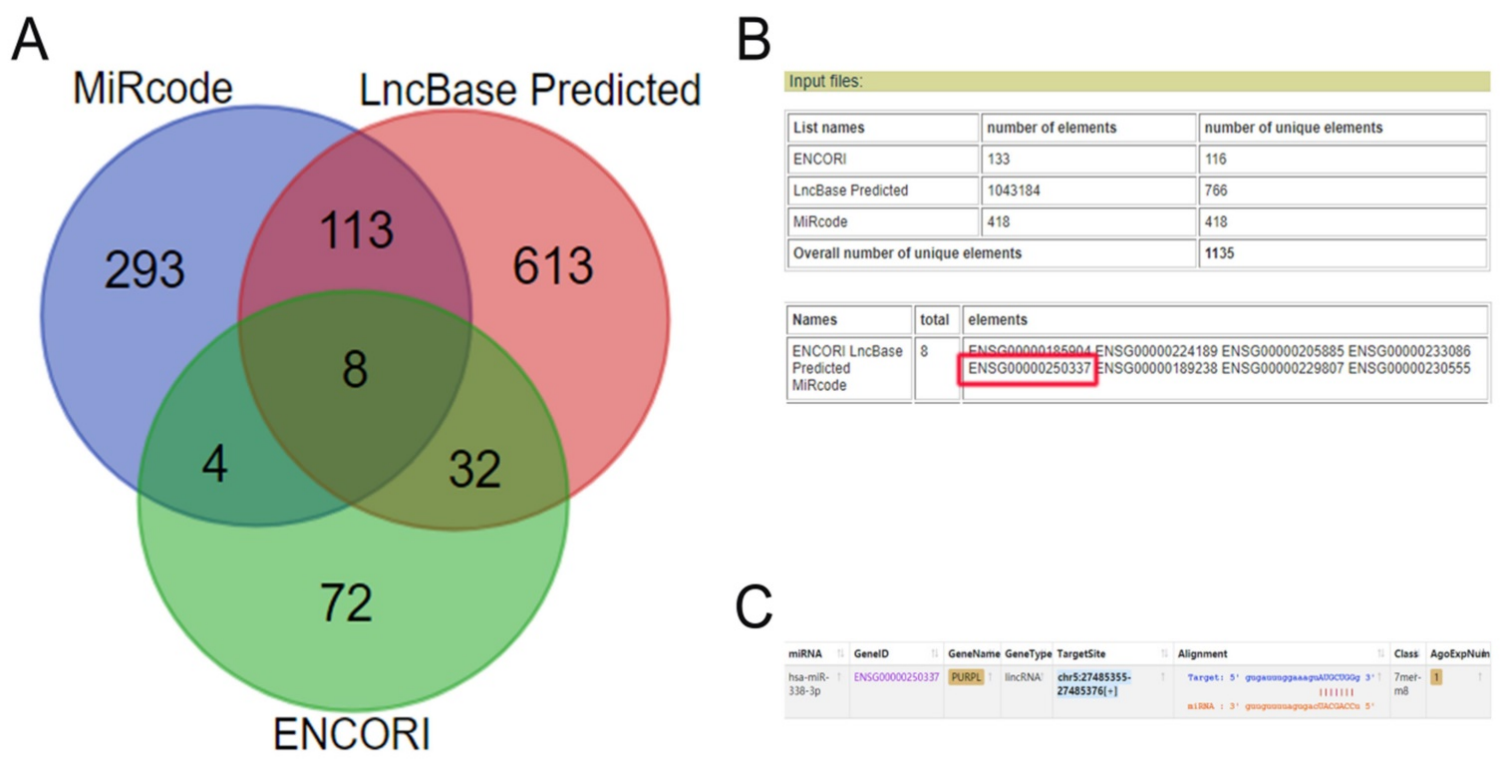
Biomedical database query predicted candidate lncRNAs targeted miR-338-3p (A.ENCORI, miRcode and LncBase Predicted databases crossed query Venn diagram for candidate lncRNAs targeted miR-338-3p; B. Input information and output result of Venn diagram producer showed PURPL (ENSG00000250337) was one of eight candidate lncRNAs targeted miR-338-3p; C. Targeted sites between PURPL and miR-338-3p indicated by ENCORI database.)

**Figure 2:**
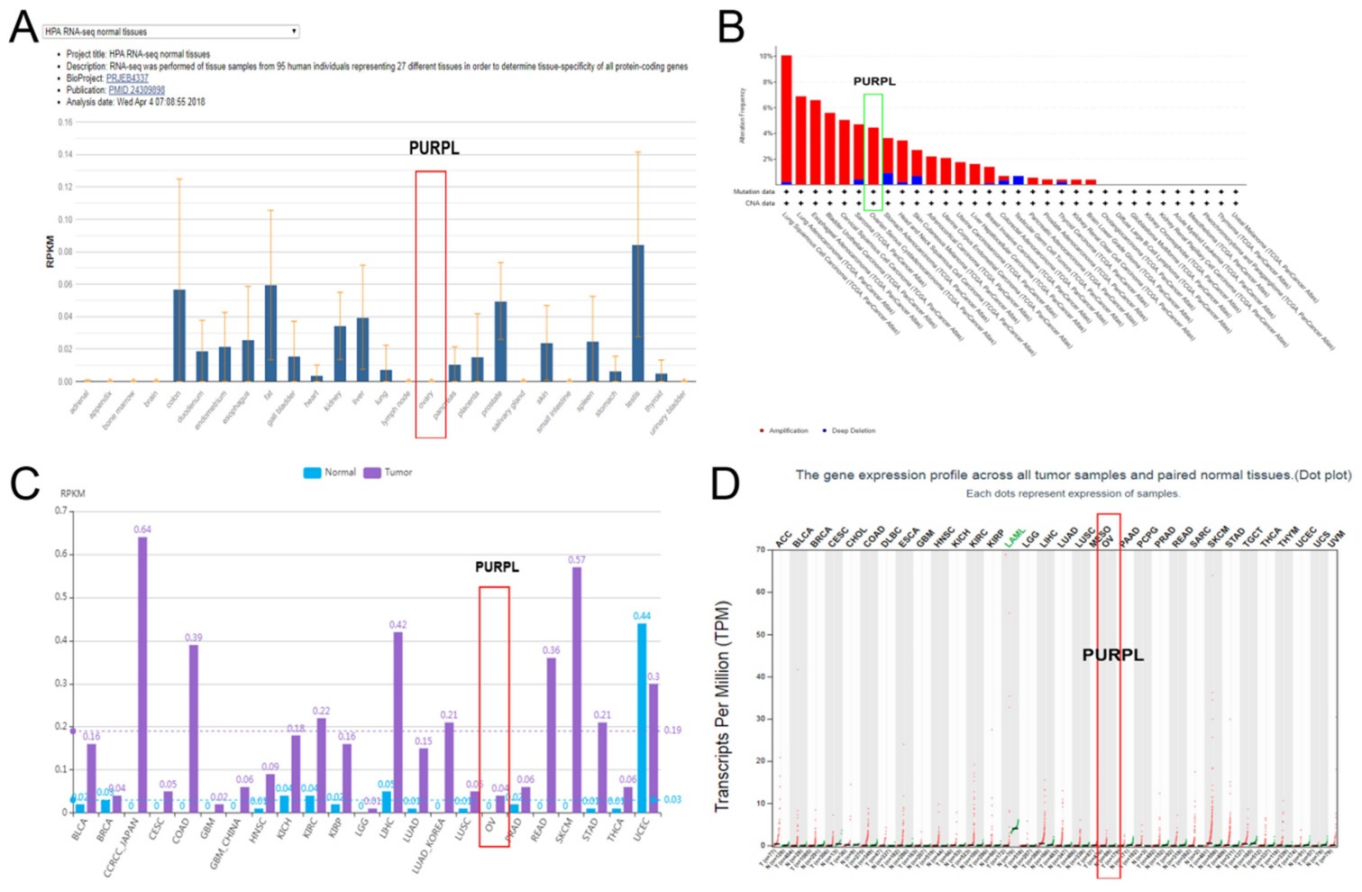
Expressions of PURPL in different human tissues queried from biomedical databases (A. Expressions of PURPL in different normal human tissues from Pubmed database; B. Expressions of PURPL in different malignant human tissues from cBioPortal database; C, D. Expressions of PURPL in paired normal ovarian and ovarian cancer tissues from lncRNASNP2 and GEPIA databases.)

Based on our previous miR-338-3p data, multiple biomedical database cross-queries showed PURPL could target miR-338-3p (Figure 1). Further queries suggested that rare expressions of PURPL were detected in normal ovarian tissues in 27 types different normal human tissue samples from 95 human individuals (Figure 2A). In 32 TCGA PanCancer Atlas studies including 10967 samples, amplifications of PURPL were detected in several malignant human tumor tissues including ovarian serous cystadenocarcinoma tissues (Figure 2B). Compared to paired normal ovarian samples, upregulated profiles of PURPL were detected in ovarian carcinoma tissues among human malignant tumors (Figure 2B and 2B).

**Figure 3:**
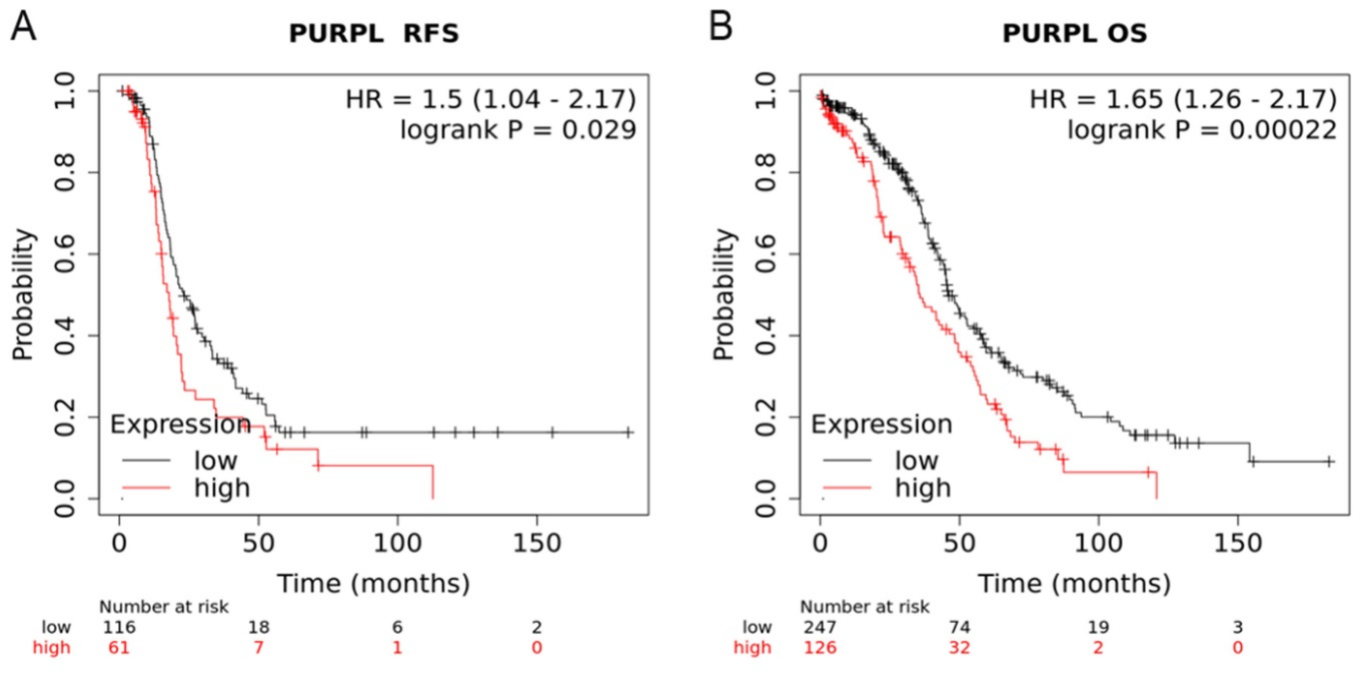
Relations of PURPL expression with the RFS and OS time of ovarian cancer from Kaplan Meier plotter database (A. RFS: recurrence free survival; B. OS: overall survival)

**Figure 4:**
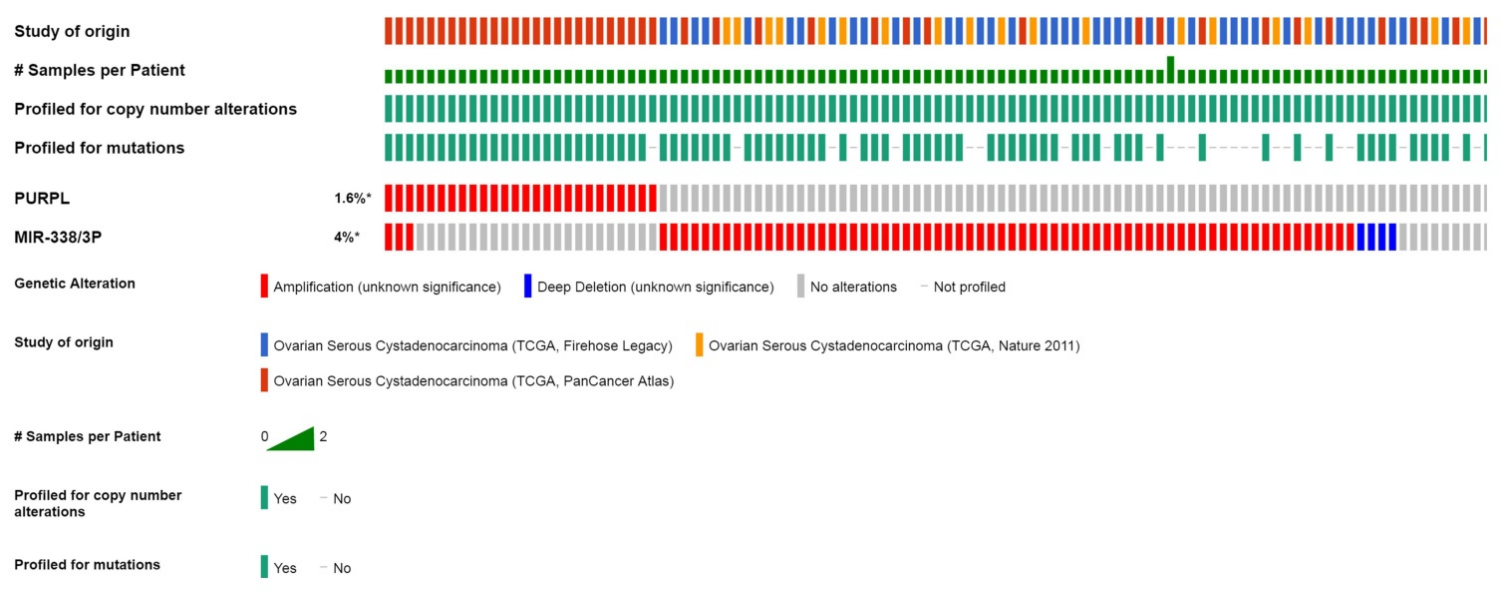
Expression profile between PURPL and miR-338-3p in ovarian cancer tissues from cBioPortal database

Compared to patients with low expression of PURPL, patients with high expression of PURPL showed shorter recurrence free survival (RFS) time and overall survival (OS) time, which was queried in Kaplan-Meier Plotter Pan-Cancer database including 374 ovarian cancer patients (Figure 3). Based on three TCGA databases including 1680 samples from 1668 ovarian cancer patients, we noted a negative expression profile between PURPL and miR-338-3p in ovarian cancer tissues (Figure 4).

### Expressions of PURPL in different collected ovarian tissues

**Figure 5:**
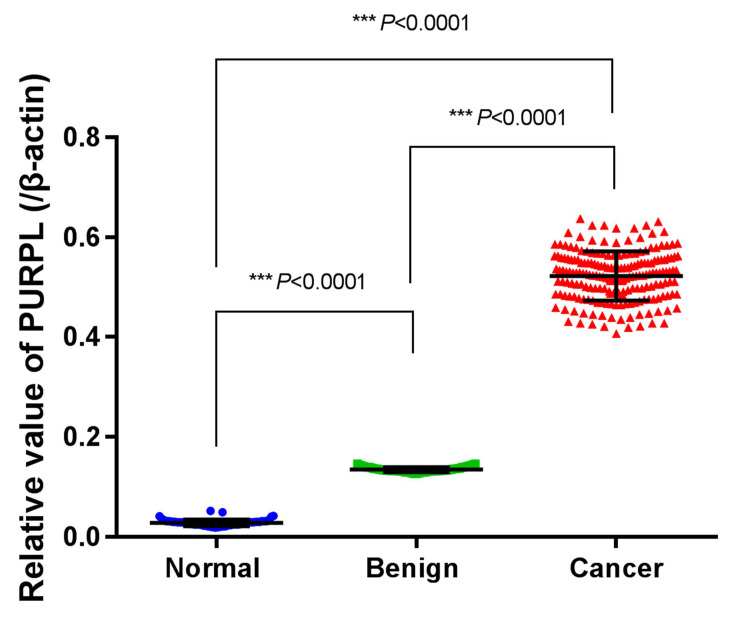
Expressions of PURPL in collected different ovarian tissues measured by real time PCR

To verify the biomedical database query results, we collected normal ovarian, benign ovarian epithelial tumor and epithelial ovarian cancer tissues to analyze the expression profile of PURPL. Low expressions of PURPL were detected in normal ovarian and benign ovarian epithelial tumor tissues. However, relative expression level of PURPL in 65 cases ovarian cancer tissues was (0.522±0.004), which indicated an obviously upregulated expression profile of PURPL in ovarian cancer tissues (F=6676.000, P<0.0001). Shown in Figure 5.

### PURPL expressions were related with clinical stage and lymph node metastasis in EOC

The median relative level of PURPL in 65 cases of epithelial ovarian cancer was (0.525±0.006), which was used to divide PURPL expression into relatively high group (higher than or equal to the median expression) and relatively low group (lower than the median expression). The expressions of PURPL were significantly higher in the EOC patients with more advanced clinical stage and developed lymph node metastasis (Table 2).

### PURPL was a high risk factor of recurrence and death for the EOC patients

Binary logistic regression analysis was used to analyze high risk factors of recurrence and death of 65 EOC patients, related factors including PURPL expression, miR-338-3p expression, age, clinical stage, histological grade, histological classification, ascites and lymph node metastasis. Upregulation of PURPL was high risk factor of the recurrence (P=0.002, OR=21.482, 95%CI: 3.457~94.251) and death (P=0.004, OR=35.643, 95%CI: 2.453~84.359) for EOC patients, other risk factors were consistent with our previous results 9.

### PURPL expression was negatively related with miR-338-3p expression in different ovarian tissues

**Figure 6:**
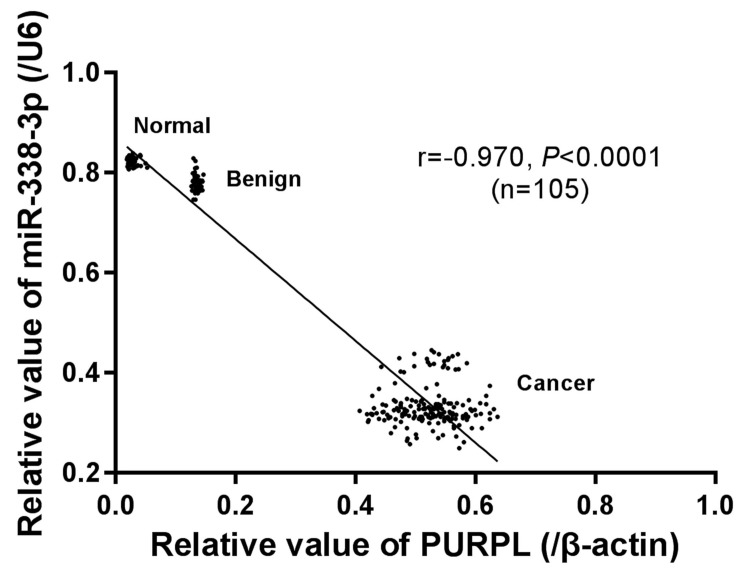
The correlation between PURPL and miR-338-3p expression in collected different ovarian tissues analyzed by Spearman correlation test

Combining our previous data, we explored the correlation between the expressions of PURPL and miR-338-3p. Analyzed by Spearman correlation test, a significant negative correlation (r = -0.970, P <0.0001) between PURPL and miR-338-3p expressions was noted in our collected 105 specimens different ovarian tissues (Figure 6).

### Upregulation of PURPL and downregulation of miR-338-3p indicated poor RFS and OS for EOC patients

**Figure 7:**
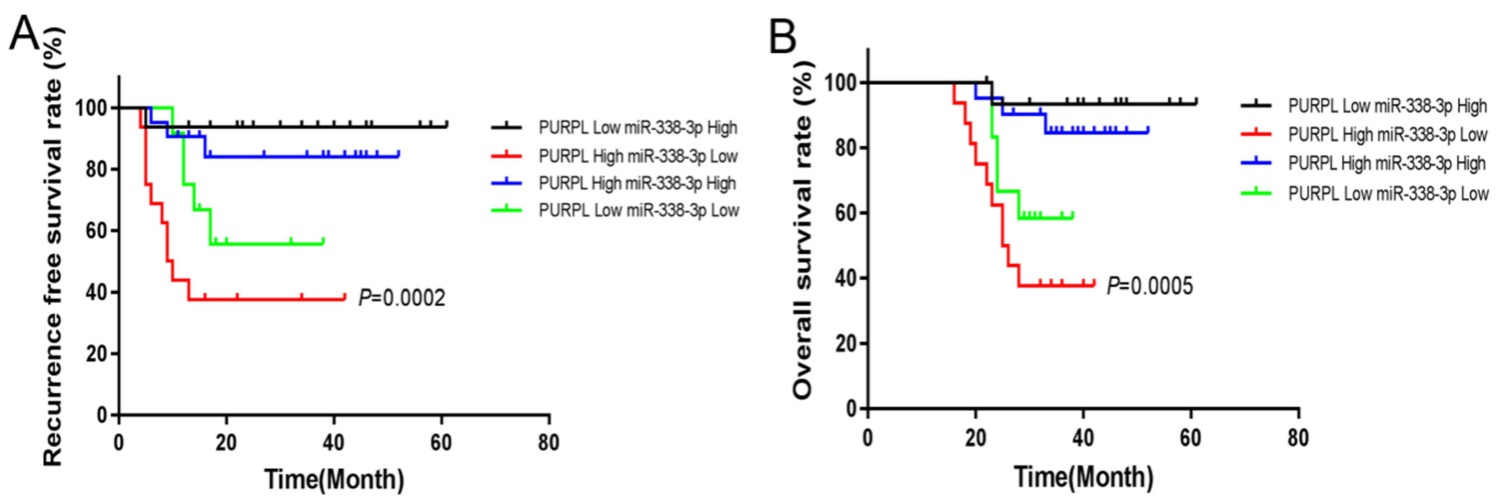
The relations between PURPL and miR-338-3p expressions and RFS and OS of EOC analyzed by Kaplan-Meier analysis

We also used the same collected data to analyze the significance of PURPL and miR-338-3p for the prognosis of EOC. The follow-up details of enrolled EOC patients were described in our previous report 9. The median expression was set to divide sub-expression groups for PURPL and miR-338-3p. The endpoints of the study were death and recurrence. Survival or no recurrence at the end of the follow-up was defined as the censored event, which was censored in statistics. At the end of the follow-up, the RFS rate of the EOC patients with high level of PURPL and low level of miR-338-3p was notably worse than other patients (χ^2^=19.410, P=0.0002), as well as the OS rate (χ^2^=17.600, P=0.0005). Shown in Figure 7.

## Discussion

Exploring the roles of noncoding RNAs in the development and progress of ovarian cancer could provide potential molecular targets and markers for clinical treatment and prognostic monitoring of ovarian cancer. MiRNAs were closely related with the development and progress of human cancers, which had been well proved. The roles of miR-338-3p disregulation in human malignancies were well discussed, such as colorectal carcinoma 17, gastric cancer 18, ovarian epithelial carcinoma 19, breast cancer 20 and lung cancer 21. The present report was an extension of our previous researches about the relations between miR-338-3p and ovarian cancer. Our reported data indicated miR-338-3p was downregulated in ovarian cancer, and involved in the growth and metastasis of ovarian cancer cells might due to the regulation of cell proliferation and EMT induced by MACC1, Met and its downstream Wnt/Catenin beta and MEK/ERK signaling pathways 9, 22.

LncRNAs played unignorable roles in human malignant tumors. LncRNAs targeted and sponged miRNAs to mediate downstream genes and signaling pathways were important regulatory mechanisms in human carcinogenesis process 23-25. In lncRNA/miRNA regulation network, different lncRNAs could target and sponge miR-338-3p to be implicated in the development and progression of human malignancies 26-29. In order to analyze the functions of lncRNAs regulated miR-338-3p in ovarian cancer cells, we used biomedical database query to filter candidate upstream lncRNAs for miR-338-3p. Multiple biomedical database cross-queries suggested PURPL could target miR-338-3p.

PURPL is about 1120bp in length, located on human chromosome 5p14.1. PURPL was reported to be involved in the development and progression of colorectal cancer, liver cancer and gastric cancer 30-32. The roles of PURPL involved in ovarian cancer have never been reported. We dug out database information about PURPL in human malignancy. PURPL was upregulated in several human cancers, including ovarian cancer. Upregulation of PURPL indicated poor RFS and OS for ovarian cancer patients. Moreover, PURPL expression showed a negative expression profile with miR-338-3p expression.

To verify the results queried in biomedical databases, we also collected normal, benign and malignant ovarian tissues to analyze the roles of PURPL in the ovarian cancer. Based on our conserved tissue specimens, expressions of PURPL were detected by real time PCR. Instead of normal ovarian tissues and benign ovarian tumor tissues, obvious upregulations of PURPL were measured in EOC tissues, which suggested abnormal expression of PURPL might be implicated in the carcinogenesis of ovarian cancer. We also analyzed the relations between PURPL expression and clinicopathological parameters of EOC. Positive relations were noted between PURPL expression and clinical stage and lymph node metastasis of EOC. Furthermore, upregulation of PURPL was a high risk factor for the recurrence and death of EOC patients. These data all indicated upregulation of PURPL might relate to poor prognosis of EOC.

Biomedical database queries suggested PURPL could target miR-338-3p, and PURPL expression shown a negative expression profile with miR-338-3p expression. Therefore, we conducted further analysis about the relation between PURPL and miR-338-3p in collected different ovarian tissues. A significantly negative relation was observed between PURPL expression and miR-338-3p expression in different ovarian tissues. We also analyzed the significances of PURPL and miR-338-3p expressions for the survival of EOC. EOC patients with high level PURPL and low level miR-338-3p presented worse RFS and OS rate than other EOC patients. These data indicated that disregulations of PURPL and miR-338-3p were implicated in the development and progression of EOC, and could serve as prognosis biomarkers for EOC.

## Conclusions

According to biomedical databases query and collected cohort tissue analysis, our present data suggested that PURPL was upregulated in EOC, PURPL expression was negatively related to miR-338-3p, and disregulations of PURPL and miR-338-3p presented poor prognosis of EOC.

## Figures and Tables

**Table 1:** Primer sequences for real time PCR

Item	Sequences (5' to 3')	Product length (bp)
PURPL	Forward:ACACGGGGCTTGAGAAATGA	376
	Reverse:TCAATCTCCAAAATAGCCGGA	
β-actin	Forward: CATGTACGTTGCTATCCAGGC	250
	Reverse: CTCCTTAATGTCACGCACGAT	

**Table 2:** The relationships between PURPL expressions in EOC tissues and clinicopathological parameters of EOC (n, %)

Item	n	PURPL		χ^2^ value	*P* value
		High	Low		
**Age**					
<50 year	31	15(48.39)	16(51.61)		
≥50 year	34	18(52.94)	16(47.06)	0.135	0.714
**FIGO stage**					
Ⅰ-Ⅱ	12	1(0.08)	11(91.67)		
Ⅲ-Ⅳ	53	31(58.49)	22(41.51)	10.785	0.001^***^
**Histological grade**					
G_3_	27	16(59.26)	11(40.74)		
G_1_-G_2_	38	15(39.47)	23(60.53)	2.477	0.116
**Histological classification**					
Serous	52	32(61.54)	20(38.46)		
Mucinous	13	6(46.15)	7(53.85)	1.014	0.314
**Ascites**					
No	16	7(43.75)	9(56.25)		
Yes	49	31(63.27)	18(36.73)	1.892	0.169
**Lymph node metastasis**					
No	33	13(39.39)	20(60.61)		
Yes	32	21(65.63)	11(34.38)	4.481	0.034^*^

## Abbreviations

lncRNAs: long noncoding RNAs
PURPL: p53 upregulated regulator of p53 levels
miR-338-3p: microRNA-338-3p
EOC: epithelial ovarian cancer
RFS: recurrence free survival
OS: overall survival
miRNAs: micro noncoding RNAs
